# Radiographic Patterns and Clinical Correlates of Medication-Related Osteonecrosis of the Jaw (MRONJ): A Retrospective Analysis †

**DOI:** 10.3390/jcm15020698

**Published:** 2026-01-15

**Authors:** Mehmet Altay Sevimay, Sedat Çetiner

**Affiliations:** Department of Oral and Maxillofacial Surgery, Faculty of Dentistry, Gazi University, 06490 Ankara, Turkey; scetiner@gazi.edu.tr

**Keywords:** osteonecrosis of the jaw, MRONJ, osteoporosis, bisphosphonates, denosumab, panoramic radiography

## Abstract

**Objectives:** This study aimed to evaluate the radiographic characteristics of medication-related osteonecrosis of the jaw (MRONJ) by digital panoramic radiographs and to investigate the associations between radiographic findings and clinical, demographic, and treatment-related variables in patients receiving antiresorptive therapy. **Methods:** A retrospective analysis was performed on 55 patients receiving antiresorptive therapy, categorized into a tooth-extraction group (*n* = 20) and an MRONJ group (*n* = 35). Standardized panoramic radiographs obtained at baseline (T0) and during the 6-month follow-up (T1) were evaluated for lamina dura thickness, trabecular bone alteration, osteosclerosis, cancellous bone loss, sequestration, and periosteal response. Statistical analyses were conducted on associations involving drug type, administration route, therapy duration, smoking, diabetes, hypertension, gender, and serum C-terminal telopeptide (CTX) levels. **Results:** The incidence of sequestrum development and cancellous bone loss was considerably higher in the MRONJ group. Sequestration demonstrated significant associations with both the duration of antiresorptive therapy (>3 years) and intermediate-risk CTX levels. No significant correlations were found between CTX and other radiographic parameters. Lamina dura thickening, trabecular alterations, osteosclerosis, and periosteal reaction exhibited no differences across groups or in relation to smoking, diabetes, age, or gender; periosteal reaction was an uncommon and variable finding. **Conclusions:** Panoramic radiography provides clinically useful information in the evaluation of MRONJ, particularly for identifying sequestration and cancellous bone degradation. The formation of sequestrum appears to be the most indicative radiographic indicator, representing both the duration of treatment and biochemical risk.

## 1. Introduction

Antiresorptive agents are extensively utilized in the management of osteoporosis, metabolic bone disorders, and malignancy-related skeletal complications due to their ability to suppress osteoclastic activity and reduce bone resorption [1,2]. In osteoporotic patients, these drugs help preserve bone mineral density and lower fracture risk, while in oncologic patients, they are employed to relieve bone pain, improve quality of life, and prevent skeletal-related events associated with multiple myeloma or metastatic involvement of solid tumors [3].

Jaw osteonecrosis was first described by Marx in 2003, who reported non-healing bone exposure in patients receiving pamidronate or zoledronate therapy; this condition was originally designated as “bisphosphonate-related osteonecrosis of the jaw” (BRONJ) [4]. As similar lesions were subsequently observed in association with other antiresorptive and antiangiogenic agents, the American Association of Oral and Maxillofacial Surgeons (AAOMS) updated the terminology to medication-related osteonecrosis of the jaw (MRONJ) in 2014 [5]. In its latest 2022 position paper, AAOMS reaffirmed the MRONJ definition, specifying three diagnostic criteria: current or previous treatment with antiresorptive or antiangiogenic drugs, no history of radiation therapy to the jaws, and exposed or probe-detectable necrotic bone in the maxillofacial region persisting for more than eight weeks [6]. Bisphosphonates and denosumab represent the most commonly prescribed agents within this therapeutic group [7].

Bisphosphonates exert their pharmacological effect by attaching to hydroxyapatite crystals in areas of active bone remodeling and inducing osteoclast apoptosis [2]. However, long-term use has been associated with impaired soft tissue healing and the development of osteonecrosis of the jaw [1]. Denosumab, a monoclonal antibody targeting the RANK/RANKL pathway, similarly reduces osteoclast differentiation and activity and has been associated with MRONJ [5].

MRONJ exhibits diverse clinical and radiographic presentations. Frequently reported radiographic features include thickening of the lamina dura, alterations in trabecular architecture, osteosclerosis, changes in cancellous bone density, sequestration, and periosteal reaction [8,9]. In clinical practice, digital panoramic radiography is commonly used as the primary imaging technique in maxillofacial evaluation due to its low cost, accessibility, and ability to visualize the entire maxillomandibular region in a single exposure [3,10].

This study aimed to characterize the radiographic characteristics of MRONJ by baseline and six-month digital panoramic radiographs and to assess their correlations with antiresorptive medication profiles and systemic factors. Specifically, changes in lamina dura thickness, alterations in trabecular bone architecture, osteosclerosis, cancellous bone destruction, sequestration, and periosteal reaction were examined in relation to drug type, route and duration of therapy, and clinical variables including smoking, diabetes, hypertension, and serum C-terminal telopeptide (CTX) levels. Through this comprehensive evaluation, the study aims to enhance understanding of the radiographic behavior of MRONJ and contribute to the development of improved diagnostic and preventive clinical strategies.

This study tests the hypothesis that the radiographic features evaluated on panoramic imaging, such as lamina dura thickening, alterations in trabecular bone architecture, osteosclerosis, cancellous bone changes, sequestrum formation, and periosteal reaction, do not exhibit significant differences between patients receiving antiresorptive therapy and those who develop MRONJ.

## 2. Materials and Methods

This research received approval from the Gazi University Ethics Committee (Decision No. E.929853 on 22 April 2024) and was conducted in accordance with the ethical principles established in the Declaration of Helsinki.

### 2.1. Patient Selection and Data Collection

In this cross-sectional study, standardized panoramic radiographs were retrospectively examined from patients who attended the Faculty of Dentistry at Gazi University between January 2021 and January 2024 and were undergoing antiresorptive treatment for osteoporosis or oncological disorders. Demographic characteristics and detailed medical history information were obtained from the university’s electronic patient management system.

The inclusion criteria for this study comprised adult patients who presented to our clinic, had utilized antiresorptive medications for a minimum of one year to manage osteoporosis or oncological conditions, and had diagnostic-quality baseline and 6-month follow-up panoramic radiographs available for assessment. All eligible individuals had complete demographic and medical records, including drug type, duration and route of administration, and systemic health information such as smoking status, diabetes mellitus, and hypertension. Serum CTX levels were recorded for patients for whom laboratory data were available to assess potential biochemical correlations with radiographic findings.

All patients received appropriate treatment protocols in accordance with AAOMS position papers, and follow-up panoramic radiographs were obtained at 6 months upon completion of the therapeutic course [5,6].

The exclusion criteria included patients with a history of radiotherapy to the head and neck region and those with metastatic bone lesions involving the jaws. Individuals previously treated surgically for MRONJ or those using antiresorptive agents for indications unrelated to osteoporosis or malignancy were excluded. To ensure the precision of radiographic evaluations, panoramic radiographs of inadequate diagnostic quality, including those with artifacts, pathological lesions, horizontal or vertical distortion, or overlapping anatomical features that impaired appropriate assessment, were excluded from the study. Additionally, patients younger than 18 years of age and scans in which the presence of periapical or periodontal inflammatory changes could bias evaluation were excluded from the study. These inflammatory changes were defined by the presence of periapical radiolucencies, loss or disruption of lamina dura continuity, widening of the periodontal ligament space, and vertical or horizontal alveolar bone loss consistent with advanced periodontal disease.

The study population was intentionally divided into two distinct groups to enable comparative radiographic evaluation. Group 1 (extraction group) consisted of 20 patients who underwent tooth extraction while receiving antiresorptive therapy and were therefore considered at risk for MRONJ; however, none of these patients fulfilled the AAOMS diagnostic criteria for MRONJ at baseline. In this group, MRONJ was not present at the initial radiographic assessment and was identified only during the follow-up period when applicable. Group 2 (MRONJ group) included 35 patients with a confirmed diagnosis of MRONJ. Accordingly, the study groups were structured to allow temporal comparison of radiographic findings and assessment of disease-related radiographic changes over time. In Group 1, the most prevalent primary condition was osteoporosis (*n* = 10), followed by lung cancer (*n* = 4), breast cancer (*n* = 2), multiple myeloma (*n* = 2). 9 patients had received intravenous (IV) zoledronic acid, 6 oral alendronic acid, 4 denosumab, and 1 IV ibandronic acid. Lesion location was distributed as follows: mandibular involvement in 11 patients, maxillary involvement in 5 patients, and both jaws in 4 patients. In Group 2, the most frequent primary diagnoses were osteoporosis (*n* = 10) and breast cancer (*n* = 7), followed by prostate cancer (*n* = 6), multiple myeloma (*n* = 4), lung cancer (*n* = 3). Among these patients, 19 had received IV zoledronic acid, 7 denosumab, 5 oral alendronic acid, 3 IV ibandronic acid, and 1 IV pamidronate. With respect to anatomical distribution, mandibular involvement was observed in 28 patients, maxillary involvement in 5 patients, and both jaws in 2 patients (Table 1).

### 2.2. Radiographic Acquisition and Evaluation

All panoramic radiographs were obtained using a single digital panoramic imaging unit (Sirona Dental Systems, Bensheim, Germany) under uniform exposure parameters, including 66 kVp, 8 mA, a 0.5 mm focal spot, and a total exposure time of 14 s, strictly adhering to the manufacturer’s positioning guidelines. Image acquisition was performed exclusively by an experienced radiology technician to ensure consistency.

The radiographs were initially saved in JPEG format (2440 × 1292 pixels, 96 dpi, 24-bit color depth) and subsequently converted to Tagged Image File Format (TIFF) to preserve optimal diagnostic quality during evaluation. All assessments were conducted in a controlled viewing environment, specifically a quiet room with dim ambient lighting, using a 15.6-inch laptop (Excalibur G770, Casper, Istanbul, Turkiye) with a 1920 × 1080 pixel resolution and a 32-bit color display.

Radiographic interpretation was performed jointly by an oral and maxillofacial radiologist and an oral and maxillofacial surgeon, both with extensive experience in MRONJ imaging, and any disagreement was resolved by consensus. To ensure the consistency of radiographic assessment, all images included in the study were re-evaluated collaboratively by the same evaluators approximately one month after the initial interpretation.

Panoramic radiographs from both Group 1 and Group 2 were systematically analyzed for the presence or absence of predefined radiographic indicators, including:

- Lamina dura thickening;
- Alterations in trabecular bone architecture;
- Osteosclerosis;
- Cancellous bone destruction;
- Sequestrum formation;
- Periosteal new bone formation.

**Figure 1:**
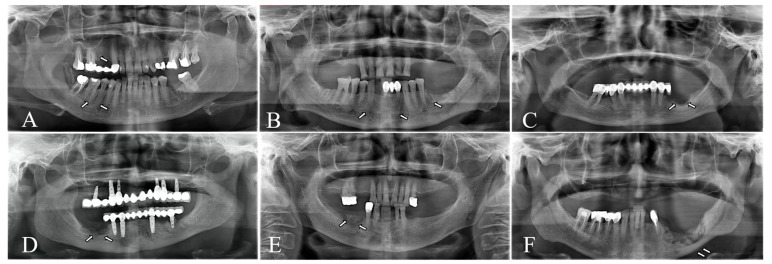
Radiographic parameters evaluated on digital panoramic radiographs. (**A**) lamina dura thickening, (**B**) trabecular bone alteration, (**C**) osteosclerosis, (**D**) cancellous bone destruction, (**E**) sequestrum formation, (**F**) periosteal new bone formation.

Each parameter was coded dichotomously (present/absent) on both baseline (T0) and 6-month follow-up (T1) radiographs, and temporal changes documented. Edentulous regions were excluded from lamina dura assessment, and periosteal reactions were not evaluated in maxillary MRONJ sites when reliable visualization was not possible. Representative radiographs illustrating these features are shown in Figure 1.

The radiographic findings were correlated with demographic variables, systemic comorbidities, characteristics of antiresorptive treatment (drug type, route, and duration), jaw site involvement, smoking status, and available CTX values to investigate potential associations with specific radiographic patterns.

### 2.3. Statistical Analysis

The required sample size was determined utilizing G*Power 3.1.9.2 (Heinrich-Heine-Universität Düsseldorf, Düsseldorf, Germany). Power analysis revealed that a minimum of 32 participants was necessary to achieve an effect size of 0.40, a Type I error rate of 0.05, a 95% confidence interval, and a statistical power of 0.80. In accordance with these parameters, 55 patients meeting the eligibility criteria were included in the study.

All statistical analyses were performed using the Statistical Package for Social Sciences (SPSS), Windows version 27 (SPSS Inc., Chicago, IL, USA). Descriptive statistics—comprising frequency, percentage, mean, standard deviation, minimum, maximum, and median values—were computed for all variables. The assumption of normality was evaluated using the Shapiro–Wilk test, and homogeneity of variances was assessed using Levene’s test. When both assumptions were met, comparisons between two independent groups were conducted using the independent samples *t*-test. When normality was violated, the non-parametric Mann–Whitney U test was applied. The chi-square test was employed to analyze associations between categorical variables. In cases where more than 20% of the anticipated cell frequencies were below five, Fisher’s exact test was used as an alternative. For all statistical analyses, the significance level (α) was set at 0.05.

## 3. Results

### 3.1. Demographic and Clinical Characteristics

A total of 55 patients were included, comprising 20 patients in Group 1 and 35 patients in Group 2.

In Group 1, osteoporosis was the most common primary condition (*n* = 10, 50.0%), followed by lung cancer (*n* = 4, 20.0%), breast cancer (*n* = 2, 10.0%), multiple myeloma (*n* = 2, 10.0%), bone cancer (*n* = 1, 5.0%), and nasopharyngeal carcinoma (*n* = 1, 5.0%). IV zoledronic acid (*n* = 9, 45.0%) and oral alendronic acid (*n* = 6, 30.0%) were the most frequently prescribed agents, whereas denosumab (*n* = 4, 20.0%) and IV ibandronate (*n* = 1, 5.0%) were less commonly used. Necrosis localization, evaluated in patients who developed postoperative complications, was predominantly in the mandible (*n* = 11, 55.0%), followed by both jaws (*n* = 5, 25.0%) and the maxilla (*n* = 4, 20.0%). Most patients were female (*n* = 13, 65.0%) and non-smokers (*n* = 19, 95.0%), while 7 patients (35.0%) presented with diabetes or hypertension. In this group, 65.0% of individuals did not develop MRONJ during follow-up.

In Group 2, osteoporosis (*n* = 10, 28.6%) and breast cancer (*n* = 7, 20.0%) were the most frequently observed underlying conditions, followed by prostate cancer (*n* = 6, 17.1%), multiple myeloma (*n* = 4, 11.4%) and lung cancer (*n* = 3, 8.6%). IV zoledronic acid constituted the majority of antiresorptive therapies (*n* = 19, 54.3%), followed by denosumab (*n* = 7, 20.0%), oral alendronic acid (*n* = 5, 14.3%), IV ibandronate (*n* = 3, 8.6%), and pamidronate (*n* = 1, 2.9%). Necrosis was most frequently localized in the mandible (*n* = 28, 80.0%), with fewer cases in the maxilla (*n* = 5, 14.3%) and both jaws (*n* = 2, 5.7%). Etiological assessment revealed that MRONJ most commonly developed following tooth extraction (*n* = 16, 45.7%), followed by peri-implantitis (*n* = 7, 20.0%), prosthetic trauma (*n* = 7, 20.0%), spontaneous onset (*n* = 4, 11.4%), and odontogenic infection (*n* = 1, 2.9%). Smoking was reported in 9 patients (25.7%), while 15 patients (42.9%) had diabetes and 19 patients (54.3%) had hypertension.

No statistically significant differences were observed between the two groups regarding mean age (*p* > 0.05, independent samples *t*-test) or gender distribution (*p* > 0.05, chi-square test).

### 3.2. Comparison of Radiographic Findings Between Groups

Changes in radiographic parameters from baseline to the 6-month follow-up were examined independently in Group 1 and Group 2 (Table 2). Chi-square and Fisher’s exact tests were employed where appropriate. A statistically significant correlation was identified between group status and the presence of “cancellous bone destruction and sequestrum formation” (*p* < 0.05), both of which were markedly more prevalent in Group 2. No significant differences were detected between the groups for lamina dura thickening, alterations in trabecular bone pattern, osteosclerosis, or periosteal reaction (*p* > 0.05). This section provides a between-group comparison of radiographic changes observed over the follow-up period and reports differences in radiographic behavior between the two clinically distinct groups.

### 3.3. Association Between Gender and Radiographic Changes

The correlation between patient gender and radiographic parameter changes was evaluated using Fisher’s Exact test (Table 3). No significant associations were identified between gender and changes in lamina dura thickness, alterations in trabecular bone, osteosclerosis, cancellous bone destruction, or sequestrum formation in either group (*p* > 0.05). However, within Group 2, a significant association was detected between gender and periosteal reaction (*p* < 0.05). All female patients (100%) demonstrated no change in periosteal appearance, whereas 4 male patients (28.6%) exhibited an increase (0 → 1) and 1 male patient (7.1%) showed a decrease (1 → 0).

### 3.4. Relationship Between Duration of Antiresorptive Therapy and Radiographic Changes

The association between treatment duration and radiographic findings was examined using chi-square and Fisher’s Exact tests (Table 4). A statistically significant relationship was found only for “sequestrum formation” (*p* = 0.020). Sequestrum formation was most frequently observed among patients who had been receiving antiresorptive therapy for over 3 years (78.6%). Conversely, patients treated for 0–3 years predominantly showed no change in sequestrum status (66.7%). No significant associations were found between treatment duration and other radiographic parameters (*p* > 0.05).

### 3.5. Association Between CTX Levels and Radiographic Findings

Radiographic alterations between T0 and T1 radiographs were compared across CTX categories (low, medium, high risk), and results are presented in Table 5. Fisher’s Exact test revealed a statistically significant correlation only for “sequestrum formation” (*p* = 0.001). In the medium-risk CTX group, sequestrum formation increased in 4 patients (44.4%), showed no change in 1 patient (11.1%), and decreased in 4 patients (44.4%), whereas individuals in the “low-risk group” showed no sequestrum increase. Other radiographic markers, lamina dura thickness, alterations in trabecular bone, osteosclerosis, cancellous bone destruction, and periosteal reaction, did not show significant associations with CTX category (*p* > 0.05).

### 3.6. Relationship Between CTX Levels and MRONJ Development in Group 1 Patients

The association between CTX levels and the clinical outcome (presence or absence of necrosis) was evaluated within Group 1 patients (Table 6). Fisher’s Exact test demonstrated a statistically significant relationship between CTX category and the progression of necrosis (*p* = 0.024). Among patients in the medium-risk CTX group, 3 patients (75.0%) developed necrosis, whereas none of the individuals in the low-risk group developed the condition.

## 4. Discussion

MRONJ is a significant complication associated with antiresorptive and antiangiogenic therapies, and its pathogenesis remains only barely explained. Multiple factors influence its development, including the type of drug administered, dosage, route of delivery, duration of therapy, and the patient’s systemic condition. Previous studies have consistently reported a substantially higher risk of MRONJ in oncologic patients receiving antiresorptive therapy compared with those treated for osteoporosis [5]. Filleul et al. emphasized that early initiation of bisphosphonate therapy in the presence of malignant bone metastasis is associated with an increased incidence of MRONJ in cancer patients, with multiple myeloma (40–50%) and breast cancer (approximately 30%) comprising the majority of cases [11].

In the present study, osteoporosis was the most common primary diagnosis in Group 1, followed by lung cancer, breast cancer, and multiple myeloma, whereas osteoporosis and breast cancer predominated in Group 2. The relatively higher proportion of osteoporosis in Group 1 compared with previous reports may reflect the inclusion of patients with long-term oral bisphosphonate therapy. In contrast, the lower prevalence of multiple myeloma in Group 2 compared with the literature suggests differences in patient demographics within the study population.

Bisphosphonates and denosumab suppress bone turnover through distinct mechanisms, and accumulating evidence suggests that the clinical and radiographic signs of MRONJ may differ based on the specific drug used. Zoledronic acid is acknowledged as the bisphosphonate associated with the highest risk of developing MRONJ [6,12]. In the literature, BRONJ is typically characterized by significant osteosclerosis, cortical bone loss, and increased bone density, whereas denosumab-related cases more frequently present with larger sequestra, more pronounced periosteal reactions, and greater marrow contrast surrounding the mandibular canal [13]. Some studies propose that denosumab-associated MRONJ follows a more aggressive clinical course [14,15], while Heim et al. and Pichardo et al. report more severe radiographic alterations in bisphosphonate-related cases [16,17]. Furthermore, the route of administration significantly influences the risk of MRONJ. Intravenous antiresorptive therapy consistently demonstrates a higher incidence compared with oral administration [18]. Anavi-Lev et al. reported that 71.2% of MRONJ cases occurred in patients receiving IV therapy, while a smaller proportion was linked to oral administration [19].

The findings of the present study correspond with these observations. IV zoledronic acid was the most commonly used agent, followed by denosumab and alendronic acid. Bisphosphonate users exhibited more frequent lamina dura thickening and osteosclerosis, whereas denosumab users demonstrated more extensive mandibular involvement and, in some cases, sequestrum formation. These results indicate that the pharmacologic characteristics of antiresorptive agents may influence the radiographic patterns of MRONJ and should be considered during clinical evaluation and treatment planning. Nevertheless, no significant radiographic differences were observed among the individual bisphosphonate formulations (zoledronic acid, alendronic acid, ibandronate, or pamidronate), suggesting that these agents may produce similar imaging.

Previous studies have shown that as the duration of antiresorptive medication exposure increases, the radiographic characteristics of MRONJ tend to become more pronounced and diverse. Shin et al. reported an average exposure duration of 58 months in osteoporotic patients and identified a clear association with increased sequestrum and higher mandibular cortical index values [20]. Similarly, Ghanaiem et al. found that extended exposure was correlated with more extensive sclerotic changes in osteoporotic patients and more significant sequestra in oncologic cases [21].

In the present study, a significant association was observed between the increased duration of antiresorptive medication exposure and sequestrum formation. Significantly, 78.6% of patients treated for over three years exhibited sequestrum progression, indicating that prolonged exposure may constitute a clinically relevant determinant of sequestrum development. In contrast, no significant associations were found between exposure duration and other radiographic features such as lamina dura thickening, trabecular pattern alteration, or osteosclerosis.

Dentoalveolar surgical procedures are the most frequently reported precipitating factors in the development of MRONJ. Filleul et al. reported that tooth extraction constituted 67% of cases, followed by spontaneous onset (26%) and prosthesis- or torus-related trauma (7%) [11]. In the study conducted by Marx et al., involving 119 patients, tooth extraction was similarly the most common trigger, followed by advanced periodontitis and periodontal surgery; notably, no identifiable etiologic factor was present in 25% of cases [22]. Collectively, these findings strongly support the role of dentoalveolar surgery, particularly tooth extraction, as an essential factor in the etiology of MRONJ. Consistently, recent AAOMS guidelines also emphasize dentoalveolar procedures, particularly tooth extraction, as the leading risk factors for MRONJ development [6].

In the present study, 45.7% of MRONJ cases were associated with previous tooth extraction. This corroborates prior literature indicating that dental surgical interventions, particularly tooth extraction, are significant triggers for MRONJ. Peri-implantitis (20%) and prosthetic trauma (20%) were the subsequent most prevalent etiological factors, while spontaneous cases accounted for 11.4%.

According to the 2022 AAOMS guidelines, advanced age (≥60 years) is recognized as an independent risk factor for MRONJ due to increased systemic comorbidities and longer durations of antiresorptive therapy [6]. MRONJ has also been reported more frequently in women, attributed to the higher prevalence of osteoporosis and breast cancer and, consequently, greater exposure to antiresorptive agents [3,9].

In the present study, however, there were no significant differences between Group 1 and Group 2 with respect to age or gender. These findings indicate differences from previous studies identifying older age and female gender as significant risk factors for MRONJ.

“Thickening of the lamina dura” is one of the most frequently reported early radiographic changes associated with antiresorptive drug use [23]. Previous studies have indicated that this finding is commonly observed in the early stages of MRONJ, whereas in more advanced stages it tends to appear alongside cortical and cancellous bone changes, sequestration, and periosteal reactions [24]. Shin et al. similarly reported that lamina dura alterations may be present in both osteoporotic and oncologic MRONJ cases, with a more severe appearance in early-stage osteoporotic patients [20].

In the present study, lamina dura thickening was observed in all patients in Group 1 at both T0 and T1, with no differences observed between intervals. A similar pattern was identified in Group 2, where lamina dura thickening was present but showed no statistically significant associations with demographic factors. These findings indicate that, while lamina dura thickening may possess diagnostic significance, it is not a dependable solitary indicator for assessing therapy response or disease progression in MRONJ.

The literature indicates that extended bisphosphonate exposure is associated with an increased frequency of trabecular osteolysis patterns, whereas osteosclerotic areas tend to become less pronounced over time. Bianchi et al. reported trabecular changes in 30 of 32 MRONJ cases evaluated using CT, and the authors emphasized that these findings can appear similar to those seen in non-healing extraction sockets [24]. Similarly, Guo et al. reported that trabecular modifications occurred predominantly in Stage 0 and Stage 1 disease and may therefore hold diagnostic value in the early phases of MRONJ [25].

In the present study, alterations in trabecular bone architecture were observed in all cases in both Group 1 and Group 2 at both T0 and T1, and this feature did not differ between the two time points. Furthermore, no substantial correlations were found between trabecular bone changes and demographic or clinical factors. Collectively, these findings suggest that although trabecular changes may assist in the radiographic diagnosis of MRONJ, they are unlikely to be reliable markers for following disease progression or treatment efficacy.

Panoramic and intraoral radiographs have demonstrated that “osteosclerosis” is an important early radiographic indicator of MRONJ [12]. Previous studies have further indicated that while osteosclerosis may emerge during the initial stages of MRONJ, its severity typically increases with disease progression. Yfanti et al. consistently recognized osteosclerosis as one of the most prevalent radiographic findings in MRONJ, accounting for approximately 75% of all features included in their radiographic index [26].

In the present study, osteosclerosis was observed in all cases in both Group 1 and Group 2 at T0 and T1 panoramic radiographs, with no interval variation between the two time points. Additionally, no significant associations were found between osteosclerosis and demographic factors. Therefore, while osteosclerosis has diagnostic significance, it is not sufficient for monitoring disease progression or treatment response and should be regarded as a supportive yet limited parameter in clinical decision-making.

“Cancellous bone destruction” is widely recognized as one of the most critical radiographic features of MRONJ. Ciofu et al. reported that cancellous bone destruction and cortical erosion were the two most frequently observed radiologic changes in MRONJ cases evaluated with Cone-Beam Computed Tomography (CBCT) [27]. Similarly, Wilde et al. demonstrated that cancellous destruction is especially common in early disease stages, such as Stage 1 [12]. In a systematic review conducted by Manole et al., cancellous bone defects were found to occur predominantly in Stage 1–2 lesions and were frequently accompanied by cortical erosion and sequestration in more advanced stages [28].

In Group 1, cancellous bone condition remained stable in 85% of cases, while 15% demonstrated new alterations during follow-up. In Group 2, 25.7% of patients exhibited resolution of previously detected cancellous destruction following treatment, while 8.6% demonstrated new destructive changes. A statistically significant difference was observed between Group 1 and Group 2 with respect to cancellous bone destruction. These results indicate that cancellous bone alteration represents a critical radiographic marker in MRONJ and should be regarded as an important parameter for evaluating both disease progression and treatment efficacy.

“Sequestration” is one of the most characteristic radiographic findings of MRONJ and reflects the separation of vital and necrotic bone. The literature indicates that sequestra are more frequently observed in advanced stages of the disease (Stages 2–3) [23]. Guo et al. identified a significant incidence of sequestration in their assessment of 16 maxillary MRONJ patients, notably in Stage 2 and Stage 3 lesions [25]. Similarly, Shin et al. found no significant difference in the presence of sequestra between osteoporotic and oncologic patient groups, although sequestra tended to be larger in oncologic cases [20].

In the present study, no significant associations were identified between sequestrum formation and demographic or systemic characteristics within Group 1 when comparing T0 and T1 panoramic radiographs. However, a statistically significant relationship was observed between the type of antiresorptive drug and sequestrum development: 57.1% of patients who developed sequestra had been treated with IV zoledronic acid, whereas 42.9% had received denosumab. This finding aligns with previous reports suggesting that both the pharmacologic profile and dosage of antiresorptive agents influence the radiographic expression of MRONJ. Additionally, a strong association was identified between clinical outcomes and sequestrum formation; all patients who developed sequestra subsequently developed necrosis, while those without necrosis exhibited no increase in sequestrum formation.

In Group 2, some patients demonstrated resolution of pre-existing sequestra following treatment, whereas others developed new sequestra that were not present at the initial evaluation. This pattern indicates that sequestration is a dynamic process influenced by both disease stage and treatment response. A statistically significant difference in sequestrum formation was also identified between Group 1 and Group 2. These findings demonstrate that sequestration, together with cancellous bone alteration, represents a critical radiographic marker in the development and assessment of MRONJ.

“Periosteal reaction” is described in the literature as a relatively uncommon radiographic finding in MRONJ, typically observed in more advanced stages. Walton et al. and Ghanaiem et al. reported comparable frequencies of lytic changes, sequestration, and periosteal reactions in osteoporotic and oncologic patients; however, both studies reported that disease severity was higher among patients treated with bisphosphonates [21,29].

The findings of the current study are consistent with these reports. In Group 1, no changes in periosteal reaction were observed between T0 and T1. In Group 2, periosteal reaction increased in 11.4% of patients, decreased in 2.9%, and remained unchanged in 85.7% between T0 and T1. No statistically significant difference in periosteal reaction was identified between the two groups.

CTX is among the most extensively utilized biochemical markers of bone resorption. Because antiresorptive therapy suppresses osteoclastic activity, CTX levels are typically reduced in treated patients [30]. Marx et al. proposed that CTX levels may be associated with MRONJ risk, categorizing values < 100 pg/mL as high risk, 100–150 pg/mL as intermediate risk, and >150 pg/mL as low risk [31]. Kwon et al. identified a relationship between CTX levels and MRONJ severity [32], whereas O’Connell et al. argued that the predictive utility of CTX is limited [33]. The research collectively indicates that CTX may function as a supporting biomarker; however, no consensus exists regarding its predictive reliability.

The present study demonstrated a substantial correlation between serum CTX levels and radiographic data specifically related to sequestrum formation. Sequestrum progression occurred in 80% of patients categorized as intermediate risk; however, no increase was recorded in those classified as low risk. Furthermore, CTX levels were significantly associated with clinical outcomes: 75% of extraction patients in the intermediate-risk category experienced necrosis at the extraction site, whereas no patients in the low-risk category exhibited necrosis. These findings align with the observations of Marx et al., supporting the idea that diminished CTX values may signify an increased vulnerability to MRONJ. However, no significant correlations were found between CTX levels and several radiographic characteristics, such as lamina dura thickness, alterations in trabecular patterns, osteosclerosis, cancellous bone destruction or periosteal reactions.

The AAOMS position papers from 2014 and 2022 identify smoking and diabetes mellitus as clinical risk factors for MRONJ; however, both emphasize that their direct associations with radiographic findings remain uncertain [5,6]. Similarly, Yoneda et al. reported smoking as a possible contributing factor without demonstrating specific radiographic correlations [34]. While diabetes has been linked to increased susceptibility and impaired healing in MRONJ, evidence supporting its influence on radiographic characteristics is limited [5,6].

Consistent with these reports, the present study found no significant associations between smoking or diabetes and changes in lamina dura thickness, trabecular pattern, osteosclerosis, cancellous bone destruction, sequestrum formation, or periosteal reaction. These findings suggest that although smoking and diabetes may contribute to MRONJ risk at a clinical level, they do not independently affect radiographic presentation. Overall, the radiographic manifestations of MRONJ appear to be multifactorial and cannot be attributed to a single systemic condition.

This study tested the hypothesis that panoramic radiographic characteristics would not significantly differ between patients receiving antiresorptive therapy and those who developed MRONJ. The findings, however, demonstrate clear radiographic differences, particularly in sequestrum formation and cancellous bone destruction, both of which showed strong associations with treatment duration and serum CTX levels and were consistently linked to clinical necrosis. These results indicate that MRONJ exhibits distinct structural alterations on panoramic imaging, thereby supporting the rejection of the null hypothesis.

Although CBCT is currently considered the gold standard for three-dimensional evaluation of MRONJ-related bone changes, panoramic radiography remains significant in routine clinical practice [6,25]. According to the AAOMS position papers, the routine use of CBCT may be limited by factors such as radiation exposure, cost, and accessibility, and should therefore be reserved for cases with specific clinical indications [6]. In contrast, panoramic radiography remains a widely available, low-dose imaging modality commonly used for initial assessment and follow-up evaluation of patients receiving antiresorptive therapy. Guo et al. demonstrated that panoramic radiography is capable of identifying major radiographic features of MRONJ, especially in moderate and advanced stages [25], while Zirk et al. emphasized its continued clinical value for routine monitoring despite the superior spatial resolution of CBCT [35].

Accordingly, while panoramic radiography cannot replace CBCT for comprehensive lesion characterization, it provides valuable information for radiographic follow-up and clinical risk evaluation in routine dental practice, in line with current AAOMS recommendations [6].

The results of this study illustrate the efficacy of panoramic radiography in evaluating MRONJ, although certain limitations must be recognized. The restricted sample size, especially within specific subgroups, could have constrained statistical power and diminished the generalizability of the findings. Although panoramic radiographs provide clinically significant information for MRONJ evaluation, reliance on a single imaging modality limits diagnostic comprehensiveness, particularly in the absence of comparative evaluation with advanced techniques such as CBCT or CT. Subsequent research incorporating multimodal imaging techniques is necessary to thoroughly validate and enhance these findings. The six-month follow-up period provides essentially a short-term perspective, which may be insufficient considering the chronic and progressive characteristics of MRONJ. Extended follow-up intervals are necessary to gain a clearer understanding of the temporal progression of radiographic alterations. Collectively, these constraints underscore the necessity for forthcoming prospective investigations using bigger sample sizes, prolonged follow-up periods, and the use of advanced imaging techniques to improve the robustness and scientific validity of the findings.

## 5. Conclusions

This study demonstrates that panoramic radiography provides significant utility in evaluating MRONJ, with sequestrum development and cancellous bone destruction identified as the most relevant radiographic findings. Sequestration demonstrated significant correlations with the duration of antiresorptive therapy and serum CTX levels, and its consistent relationship with necrosis emphasizes its significance as a parameter for evaluating disease progression. Conversely, CTX levels had no association with further radiographic characteristics, such as lamina dura thickening, trabecular alterations, osteosclerosis, or periosteal reaction. Smoking and diabetes were identified as clinical risk factors in the literature; however, in this study, they did not independently affect radiographic patterns, and age and gender also had no significant impact.

## Figures and Tables

**Table 1:** Demographic and clinical characteristics of patients receiving antiresorptive therapy.

	Group 1 Tooth-Extraction Group (*n* = 20)	Group 2 MRONJ Group (*n* = 35)
*Age (Range, Mean ± SD)* *Gender*	62 (41–87) 60.45 ± 11.33	64 (43–85) 63.63 ± 10.59
-Male	7 cases (35%)	14 cases (40%)
-Female	13 cases (65%)	21 cases (60%)
*Primary Disease*		
-Breast cancer	2 cases (10%)	7 cases (20%)
-Lung cancer	4 cases (20%)	3 cases (8.6%)
-Prostate cancer		6 cases (17.1%)
-Multiple myeloma	2 cases (10%)	4 cases (11.4%)
-Nasopharyngeal cancer	1 case (5%)	
-Ovarian cancer		1 case (2.9%)
-Liver cancer		1 case (2.9%)
-Renal cancer		1 case (2.9%)
-Tongue cancer		1 case (2.9%)
-Bone cancer	1 case (5%)	1 case (2.9%)
-Osteoporosis	10 cases (50%)	10 cases (28.6%)
*Type of Antiresorptive* *Medication*	Zoledronic Acid: 9 cases (45%) Denosumab: 4 cases (20%) Ibandronic Acid: 1 case (30%)	Zoledronic Acid: 19 cases (54.3%) Denosumab: 7 cases (20%) Ibandronic Acid: 3 cases (8.6%) Pamidronate: 1 case (2.9%) Alendronic Acid: 5 cases (14.3%)
*Route of Administration*		
-Intravenous	10 cases (50%)	23 cases (65.7%)
-Oral	6 cases (30%)	5 cases (14.3%)
-Subcutaneous	4 cases (20%)	7 cases (20%)
*Duration of Antiresorptive* *Therapy (Range, Mean ± SD)*	2 years (1–8), 3.2 ± 2.4	4 years (1–15), 4.6 ± 3.01
*Lesion Location*	Mandible: 11 cases (55%) Maxilla: 4 cases (20%) Both jaws: 5 cases (25%)	Mandible: 28 cases (80%) Maxilla: 5 cases (14.3%) Both jaws: 2 cases (5.7%)
*Smoking Status*	Yes: 1 case (5%) No: 19 cases (95%)	Yes: 9 cases (25.7%) No: 26 cases (74.3%)
*Diabetes Mellitus*	Yes: 7 cases (35%) No: 13 cases (65%)	Yes: 15 cases (42.9%) No: 20 cases (57.1%)
*Hypertension*	Yes: 7 cases (35%) No: 13 cases (65%)	Yes: 19 cases (54.3%) No: 16 cases (45.7%)

**Table 2:** Comparison of T0–T1 radiographic findings between Group 1 and Group 2.

		Group							
		Tooth-Extraction Group		MRONJ Group		Total			
		*n*	%	*n*	%	*n*	%	Test Statistic	*p*
Lamina Dura Thickening	Increased (0 → 1)	0	0.0	4	11.4	4	7.3	2.704	0.285
	No change	20	100.0	30	85.7	50	90.9		
	Decreased (1 → 0)	0	0.0	1	2.9	1	1.8		
Trabecular Bone Pattern Alteration	Increased (0 → 1)	0	0.0	0	0.0	0	0.0	-	-
	No change	20	100.0	35	100.0	55	100.0		
	Decreased (1 → 0)	0	0.0	0	0.0	0	0.0		
Osteosclerosis	Increased (0 → 1)	1	5.0	0	0.0	1	1.8	-	0.364
	No change	19	95.0	35	100.0	54	98.2		
	Decreased (1 → 0)	0	0.0	0	0.0	0	0.0		
Cancellous Bone Destruction	Increased (0 → 1)	3	15.0	3	8.6	6	10.9	6.883	0.032 *
	No change	17	85.0	23	65.7	40	72.7		
	Decreased (1 → 0)	0	0.0	9	25.7	9	16.4		
Sequestrum Formation	Increased (0 → 1)	7	35.0	7	20.0	14	25.5	χ^2^ = 12.486	0.002 *
	No change	13	65.0	14	40.0	27	49.1		
	Decreased (1 → 0)	0	0.0	14	40.0	14	25.5		
Periosteal New Bone Formation	Increased (0 → 1)	0	0.0	4	11.4	4	7.3	2.704	0.285
	No change	20	100.0	30	85.7	50	90.9		
	Decreased (1 → 0)	0	0.0	1	2.9	1	1.8		

* *p* < 0.05 indicates statistical significance. χ^2^: Chi-square test statistic.

**Table 3:** Comparison of radiographic findings by gender in Group 1 and Group 2.

		Group											
		Tooth-Extraction Group						MRONJ Group					
		Gender						Gender					
		Female		Male		Test Statistic	*p*	Female		Male		Test Statistic	*p*
		*n*	%	*n*	%			*n*	%	*n*	%		
Lamina Dura Thickening	Increased (0 → 1)	0	0.0	0	0.0	-	-	1	4.8	3	21.4	2.671	0.279
	No change	13	100.0	7	100.0			19	90.5	11	78.6		
	Decreased (1 → 0)	0	0.0	0	0.0			1	4.8	0	0.0		
Trabecular Bone Pattern Alteration	Increased (0 → 1)	0	0.0	0	0.0	-	-	0	0.0	0	0.0	-	-
	No change	13	100.0	7	100.0			21	100.0	14	100.0		
	Decreased (1 → 0)	0	0.0	0	0.0			0	0.0	0	0.0		
Osteosclerosis	Increased (0 → 1)	0	0.0	1	14.3	-	0.350	0	0.0	0	0.0	-	-
	No change	13	100.0	6	85.7			21	100.0	14	100.0		
	Decreased (1 → 0)	0	0.0	0	0.0			0	0.0	0	0.0		
Cancellous Bone Destruction	Increased (0 → 1)	1	7.7	2	28.6	-	0.270	3	14.3	0	0.0	2.336	0.316
	No change	12	92.3	5	71.4			12	57.1	11	78.6		
	Decreased (1 → 0)	0	0.0	0	0.0			6	28.6	3	21.4		
Sequestrum Formation	Increased (0 → 1)	3	23.1	4	57.1	-	0.174	5	23.8	2	14.3	5.552	0.078
	No change	10	76.9	3	42.9			5	23.8	9	64.3		
	Decreased (1 → 0)	0	0.0	0	0.0			11	52.4	3	21.4		
Periosteal New Bone Formation	Increased (0 → 1)	0	0.0	0	0.0	-	-	0	0.0	4	28.6	8.123	0.006 *
	No change	13	100.0	7	100.0			21	100.0	9	64.3		
	Decreased (1 → 0)	0	0.0	0	0.0			0	0.0	1	7.1		

* *p* < 0.05 indicates statistical significance.

**Table 4:** Changes between the T0–T1 radiographs in relation to the duration of antiresorptive therapy.

		Duration of Antiresorptive Therapy							
		0–3 Years		More than 3 Years		Total			
		*n*	%	*n*	%	*n*	%	Test Statistic	*p*
Lamina Dura Thickening	Increased (0 → 1)	3	11.1	1	3.6	4	7.3	1.932	0.479
	No change	24	88.9	26	92.9	50	90.9		
	Decreased (1 → 0)	0	0.0	1	3.6	1	1.8		
	Total	27	100.0	28	100.0	55	100.0		
Trabecular Bone Pattern Alteration	Increased (0 → 1)	0	0.0	0	0.0	0	0.0	-	-
	No change	27	100.0	28	100.0	55	100.0		
	Decreased (1 → 0)	0	0.0	0	0.0	0	0.0		
	Total	27	100.0	28	100.0	55	100.0		
Osteosclerosis	Increased (0 → 1)	1	3.7	0	0.0	1	1.8	-	0.491
	No change	26	96.3	28	100.0	54	98.2		
	Decreased (1 → 0)	0	0.0	0	0.0	0	0.0		
	Total	27	100.0	28	100.0	55	100.0		
Cancellous Bone Destruction	Increased (0 → 1)	4	14.8	2	7.1	6	10.9	1.191	0.567
	No change	18	66.7	22	78.6	40	72.7		
	Decreased (1 → 0)	5	18.5	4	14.3	9	16.4		
	Total	27	100.0	28	100.0	55	100.0		
Sequestrum Formation	Increased (0 → 1)	3	11.1	11	39.3	14	25.5	χ^2^ = 7.842	0.020 *
	No change	18	66.7	9	32.1	27	49.1		
	Decreased (1 → 0)	6	22.2	8	28.6	14	25.5		
	Total	27	100.0	28	100.0	55	100.0		
Periosteal New Bone Formation	Increased (0 → 1)	1	3.7	3	10.7	4	7.3	1.853	0.611
	No change	25	92.6	25	89.3	50	90.9		
	Decreased (1 → 0)	1	3.7	0	0.0	1	1.8		
	Total	27	100.0	28	100.0	55	100.0		

* *p* < 0.05 indicates statistical significance. χ^2^: Chi-square test statistic.

**Table 5:** Distribution of radiographic parameter changes (T0–T1) according to CTX levels.

		CTX Levels									
		High Risk		Intermediate Risk		Low Risk		Total			
		*n*	%	*n*	%	*n*	%	*n*	%	Test Statistic	*p*
Lamina Dura Thickening	Increased (0 → 1)	0	0.0	0	0.0	1	9.1	1	4.8	4.605	1.000
	No change	1	100.0	9	100.0	9	81.8	19	90.5		
	Decreased (1 → 0)	0	0.0	0	0.0	1	9.1	1	4.8		
	Total	1	100.0	9	100.0	11	100.0	21	100.0		
Trabecular Bone Pattern Alteration	Increased (0 → 1)	0	0.0	0	0.0	0	0.0	0	0.0	-	-
	No change	1	100.0	9	100.0	11	100.0	21	100.0		
	Decreased (1 → 0)	0	0.0	0	0.0	0	0.0	0	0.0		
	Total	1	100.0	9	100.0	11	100.0	21	100.0		
Osteosclerosis	Increased (0 → 1)	0	0.0	0	0.0	0	0.0	0	0.0	-	-
	No change	1	100.0	9	100.0	11	100.0	21	100.0		
	Decreased (1 → 0)	0	0.0	0	0.0	0	0.0	0	0.0		
	Total	1	100.0	9	100.0	11	100.0	21	100.0		
Cancellous Bone Destruction	Increased (0 → 1)	0	0.0	1	11.1	1	9.1	2	9.5	3.435	1.000
	No change	1	100.0	8	88.9	9	81.8	18	85.7		
	Decreased (1 → 0)	0	0.0	0	0.0	1	9.1	1	4.8		
	Total	1	100.0	9	100.0	11	100.0	21	100.0		
Sequestrum Formation	Increased (0 → 1)	1	100.0	4	44.4	0	0.0	5	23.8	13.319	0.001 *
	No change	0	0.0	1	11.1	9	81.8	10	47.6		
	Decreased (1 → 0)	0	0.0	4	44.4	2	18.2	6	28.6		
	Total	1	100.0	9	100.0	11	100.0	21	100.0		
Periosteal New Bone Formation	Increased (0 → 1)	0	0.0	0	0.0	0	0.0	0	0.0	-	-
	No change	1	100.0	9	100.0	11	100.0	21	100.0		
	Decreased (1 → 0)	0	0.0	0	0.0	0	0.0	0	0.0		
	Total	1	100.0	9	100.0	11	100.0	21	100.0		

* *p* < 0.05 was considered statistically significant.

**Table 6:** Relationship Between CTX Levels and Treatment Outcome in Group 1.

Sequestrum Formation	CTX Levels	Treatment Outcome							
		No Necrosis		Necrosis Developed		Total			
		*n*	%	*n*	%	*n*	%	Test Statistic	*p*
Increased (0 → 1)	High Risk	0	0.0	0	0.0	0	0.0	-	-
	Intermediate Risk	0	0.0	3	100.0	3	100.0		
	Low Risk	0	0.0	0	0.0	0	0.0		
	Total	0	0.0	3	100.0	3	100.0		
No change	High Risk	0	0.0	0	0.0	0	0.0	-	-
	Intermediate Risk	1	12.5	0	0.0	1	12.5		
	Low Risk	7	87.5	0	0.0	7	87.5		
	Total	8	100.0	0	0.0	8	100.0		
Total	High Risk	0	0.0	0	0.0	0	0.0	-	0.024 *
	Intermediate Risk	1	12.5	3	100.0	4	36.4		
	Low Risk	7	87.5	0	0.0	7	63.6		
	Total	8	100.0	3	100.0	11	100.0		

* *p* < 0.05 was considered statistically significant.

## Data Availability

The data presented in this study are available on request from the corresponding author due to privacy and ethical restrictions, as the dataset contains patient information that cannot be shared publicly.

## Abbreviations

The following abbreviations are used in this manuscript:

MRONJ	Medication-Related Osteonecrosis of the Jaw
CTX	C-terminal telopeptide
BRONJ	Bisphosphonate-Related Osteonecrosis of the Jaw
AAOMS	American Association of Oral and Maxillofacial Surgeons
TIFF	Tagged Image File Format
IV	Intravenous
CBCT	Cone-Beam Computed Tomography
T0	Baseline
T1	6-month follow-up
